# Supplementary material for: Human movement and environmental barriers shape the emergence of dengue
Source: Nat Commun. 2024 May 28;15:4205. doi: 10.1038/s41467-024-48465-0 (PMC11133396; doi:10.1038/s41467-024-48465-0)
Supplement: Supplementary file 1 — Supplementary information [file 41467_2024_48465_MOESM1_ESM.pdf]

# Human movement and environmental barriers constrain past, present, and future spread of dengue in Brazil and Mexico

## Supplementary Information

## Table of Contents


**1.1 Human movement models and data processing ........ 2**
Great circle distance ........ 2
Gravity and radiation models ........ 2
Adjacency ........ 2
Flight data ........ 3
Internal migration ........ 3
Travel time ........ 3
Movement data processing ........ 4

**1.2 Geospatial model hyperparameters ........ 5**

**1.3 Historical sources of infection for Brazil ........ 7**
Sources from case report data ........ 7
Sources from genetic data and phylogeographic analysis ........ 7

**1.4 Historical and future projection of climate and mobility features ........ 10**

***SI Figure 1 – Optimal thresholds for defining invasion. ........ 12***

***SI Figure 2- Expanding window time-series cross validation of the survival model in Brazil (A and B) and Mexico (C and D). ........ 13***

***SI figure 3 – Geospatial model selection heatmap. ........ 14***

***SI Figure 4 Geospatial model predictive performance over time. ........ 15***

***SI Figure 5 – Variable importance ........ 16***

***SI Figure 6 – Geospatial model country cross-validation. ........ 17***

***SI Figure 7 – Possible origins of dengue spread in Brazil from sporadic outbreak reports. ........ 18***

***SI Figure 8 – Projection of model features in keeping with climate scenario. ........ 19***

**References ........ 20**

## 1.1 Human movement models and data processing

### Great circle distance

The geodesic distance between municipality centroids was calculated using the “distm” function in the *geosphere* R package.

### Gravity and radiation models

Human movement between municipality $i$ and municipality $j$ ($M_{i\to j}$) is calculated using a standard parameterised gravity and radiation models:

Gravity:

$$M_{i\to j} = \theta_G \frac{{p_i}^{\alpha} {p_j}^{\beta}}{{d_{i\to j}}^{\gamma}}$$

Radiation:

$$M_{i\to j} = \theta_R \frac{p_i p_j}{(p_i + s_{ij})(p_i + p_j + s_{ij})}$$

where:

$d_{i\to j}$ = The geodesic distance (i.e. ignoring water bodies) between centroids of municipalities $i$ and $j$

$p_i$ = population of municipality $i$

$s_{ij}$= total population living in municipalities whose centroid falls within the distance $d_{i\to j}$ of the centroid of patch $i$

$\alpha, \beta, \gamma, \theta$ = scaling parameters

We choose the following scaling parameter values based on the fit of this gravity model to high resolution call data records that has previous been shown to generalise well across settings[1]. This model formulation and parameterisation has previously been shown to accurately represent human movement in a variety of international settings and predict the spread of infectious diseases and their mosquito vectors [2,3].

| Parameter | Value |
|---|---|
| $\alpha$ | 0.44 |
| $\beta$ | 0.4 |
| $\gamma$ | 1.81 |
| $\theta_G$ | 0.01 |
| $\theta_R$ | 0.9 |

**Table SI 1. Gravity model parameters.**

### Adjacency

A binary adjacency matrix between municipalities was calculated using the “poly2nb” function in the *spdep* R package. Elements in this matrix take the value of 1 if municipalities share a border and 0 if they do not.

## Flight data

Airline ticket sales data was extracted from the Global database of the International Air Transport Association (www.iata.org) to estimate domestic passenger numbers between all airport combinations in Mexico and Brazil. The total number of registered tickets between cities from January 2009 to December 2019 were aggregated to provide an estimate of the volume of people moving between each airport pair.

To distribute traffic flows from airports to municipalities we follow Huber et al. [4] in using a Huff model. The probability of a resident living in municipality $i$ flying from airport $j$ is a function of the attractiveness of airport $j$ and the distance between the centroid of municipality $i$ and airport $j$. Airport attractiveness is estimated by total outbound passenger volume and, as in [4], we choose a distance exponent of ($\beta = 2$) and a maximum distance cut off of 500km to limit the catchment size of ground-based travel to and from the airport:

$$P_{ij} = \frac{S_j / D_{ij}^{\beta}}{\sum_{i=1}^{n} S_j / D_{ij}^{\beta}}$$

To estimate the relative number of travellers between municipalities we need to take into account the resident population ($R_i$) of each municipality and the range of airport options open to them:

$$T_{ij} = \frac{R_i P_{ij}}{\sum_{j=1}^{n} P_{ij}}$$

The relative distribution of passengers from any given airport across municipalities in their catchment areas can therefore be given by:

$$D_{ij} = \frac{T_{ij}}{\sum_{i=1}^{n} T_{ij}}$$

Which we use to distribute origin-destination traveller numbers between pairs of municipalities.

## Internal migration

We used 2010, 5 year estimated internal migration flows from WorldPop [5] to characterise long term movement patterns. These datasets were only available at the state (admin 1) level, so were downscaled to municipality level assuming equal proportional flows between municipalities in each state.

## Travel time

We use combine a global travel time “friction surface”[6] with minimum route finding algorithms[7] to derive a point estimate of travel time by motorised transport between the most densely populated pixels in each municipality.

## Movement data processing

To measure the association between risk of dengue spread and a range of connectivity metrics data transformations were applied to different human movement datasets to improve parameters estimates and interpretability at the modelling stage (Table SI 1).

| **Feature** | **Transformation** |
|---|---|
| Great circle distance | $\log\left(\frac{1}{x}\right)$ |
| Gravity model | $\log(x+1)$ |
| Radiation model | $\log(x+1)$ |
| Adjacency | $x$ |
| Flight | $\log(x+1)$ |
| Internal migration | $\log(x+1)$ |
| Travel time | $\log\left(\frac{1}{x}\right)$ |

**Table SI 2. Transformation of movement features.**

## 1.2 Geospatial model hyperparameters

| Model | Hyperparameters Tuned |
|---|---|
| Logistic regression (*glm*) | NA |
| Lasso regression (*glmnet*) | ***Lambda*** – Range (log-10 transformed scale): [-10, 0] |
| Ridge regression (*glmnet*) | ***Lambda*** – Range (log-10 transformed scale): [-10, 0] |
| Elastic net (*glmnet*) | ***Lambda*** – Range (log-10 transformed scale): [-10, 0];<br>***Alpha***: Range [0, 1] |
| Decision tree (*rpart*) | ***Cost_Complexity*** – Range (log-10 transformed scale): [-10, -1];<br>***Tree_Depth*** – Range: [1, 15];<br>***Min_N*** – Range: [2, 40] |
| Random forest (*ranger*) | ***MTry*** – Range: [1, 16];<br>***Min_N*** – Range: [2, 40];<br>***Trees*** – Range: [1, 2000] |
| Gradient boosted decision trees (*XGBoost*) | ***Min_N*** – Range[2, 40];<br>***Tree_Depth*** – Range[1, 15];<br>***Learn_Rate*** – Range (log-10 transformed scale): [-10, -1];<br>***Loss_Reduction*** – Range (log-10 transformed scale): [-10, -1.5];<br>***Trees*** – Range[1, 2000];<br>***Sample_Size*** – Range: [0.5, 0.99];<br>***Stop_Iter*** – 8 (fixed);<br>***MTry*** – Range: [1,16] |
| K-nearest neighbours (*kknn*) | ***Neighbours*** – Range: [3,5,7,9, 17, 33, 69, 97, 183, 211] |
| Multilayer perceptron (*keras*) | ***Hidden_Units*** – Range[1, 10];<br>***Dropout*** – Range[0, 1];<br>***Penalty*** – Range (log-10 transformed scale): [-10, 0];<br>***Learn_Rate*** – Range (log-10 transformed scale): [-10, -1];<br>***Epochs*** – left as default (20);<br>***Activation*** – left as default (softmax) |

**Table SI 3. Hyperparameters tuned during geospatial model selection.** Each model was tested over the same 5 random seeds, with the training set (75% of full dataset) split into five-folds for cross-validation for hyperparameter tuning. All hyperparameters were explored over their default ranges in the *Dials* package, except for KNN. Maximum entropy grids of between 25 and 125 combinations (5 per parameter when there are multiple parameters to tune up until 3 due to computational constraints were tested) of hyperparameters were explored during grid searches. Classification thresholds (between 0 and 0.99 by increments of 0.01)) were set to minimize the difference between specificity and sensitivity in the training set.

| Country | XGBoost Hyperparameter Values |
| --- | --- |
| Mexico | ***Min_N*** – 4;<br>***Tree_Depth*** – 14;<br>***Learn_Rate*** – 0.04653296;<br>***Loss_Reduction*** – 6.311998e-10;<br>***Trees*** – 1623;<br>***Sample_Size*** – 0.7782342;<br>***Stop_Iter*** – 15;<br>***MTry*** – 8 |
| Brazil | ***Min_N*** – 3;<br>***Tree_Depth*** – 15;<br>***Learn_Rate*** – 0.03023025;<br>***Loss_Reduction*** – 0.05793492;<br>***Trees*** – 1495;<br>***Sample_Size*** – 0.8824715;<br>***Stop_Iter*** – 18;<br>***MTry*** – 15; |

**Table SI 4. Hyperparameters for XGBoost models used in experiments.** Once XGBoost was selected as the geospatial model of choice, hyperparameters were trained in 10-fold cross validation using a 75% training set with maximum entropy grids of up to 125 different combinations of hyperparameter values (due to computational constraints). These were the hyperparameters subsequently used across experiments (e.g. across time-series cross validation, historical reconstruction, future projection) in combination with the temporal model for thresholding.

## 1.3 Historical sources of infection for Brazil

### Sources from case report data

To identify possible origins of pre-2000 dengue spread in Brazil we examined a global dengue occurrence database assembled by Messina and colleagues detailing occurrence records between 1960 and 2012[8]. This database collates records of dengue occurrence from peer-reviewed literature and case reports and informal online sources. As an opportunistic sample of dengue occurrence (i.e. not incidence) this database is not comparable to the case database from 2001 onwards in Brazil and cannot be used to infer patterns of spread, however it can be useful for identifying possible origins. Occurrence records in Brazil and neighbouring countries between 1986 (first Brazilian record in this database) and 2001 (the beginning of our case dataset for Brazil) are displayed in SI Figure 6. It is noted that an earlier outbreak did occur in Boa Vista in 1981/82, but as this did not lead to ongoing spread, it was excluded as a possible source of wider spread[9,10]. Based on a visual inspection of geographic trends over time we identified four potential geographically unique introductions that are more likely to have occurred via international than domestic spread. These included the initial outbreaks in Fortaleza and Rio de Janeiro in 1986 that continue to persist and spread within their respective regions in all subsequent years. We also propose a novel geographic introduction into northern Sao Paulo state as the cause of the 1990 epidemic with the largest town in the region (Ribeiro Preto as the chosen source for our analysis). While Ribeiro Preto is relatively geographically close to Rio de Janeiro in the Brazilian context, the absence of reported cases in many larger climatically suitable cities closer to Rio de Janeiro led us to believe that a potential international introduction to Ribeiro Preto in 1990 was worth testing in our spread model simulations. Finally, an independent international introduction to the Amazonian city of Manaus in 1996 was proposed based on regular reporting of cases in countries to the North of Brazil (particularly Venezuela and French Guiana) since 1990, Manaus's role as a regional hub for international river-based traffic and the occurrence of dengue in many rural areas in the east of the country post 1996 that could not easily be explained without a source in Manaus.

### Sources from genetic data and phylogeographic analysis

To investigate the evolutionary origins of dengue virus lineages in Brazil, we analysed publicly available serotype-specific dengue virus genetic datasets using spatiotemporal phylogenetic approaches. First, we downloaded complete and near complete dengue virus sequences (≥8000 base pairs, bp) from GenBank/NCBI [11](28 June 2022). Many of the earlier sequences from Brazil correspond to the E gene codifying for the envelop protein (1485 bp). Thus, to capture earlier dengue lineages circulating in Brazil, we also downloaded all sequences from Brazil with length ≥1000 bp. We removed identical sequences and sequences without information on country of origin, Brazilian region of origin (for Brazilian sequences only), or year of collection.

The number of assembled dengue sequences was as follows: DENV1 (*n*=3998 sequences), DENV2 (*n*=3441), DENV3 (*n*=1782) and DENV4 (*n*=1223). Sequence alignments were performed with minimap2 v2.24 [12] and gofasta v1.1.0 [13], using DENV NCBI RefSeq genomes as references. Untranslated genomic regions were trimmed from the alignments. Maximum-likelihood (ML) phylogenetic trees were then inferred from each alignment with IQ-Tree v2.1.2 [14], under the

GTR+F+I+G4 model [15,16]. Shimoidara-Hasegawa like approximate likelihood ratio test [17] was used to estimate branch statistical support.

From the ML trees, we identified the clades corresponding to the genotypes that have been identified so far in Brazil: DENV1 genotype I (DENV1-I); DENV2 genotype III (DENV2-III); DENV3 genotype III (DENV3-III); and DENV4 genotype II (DENV4-II). Separate datasets for each of these genotypes were then assembled, and novel ML phylogenies were inferred as described above. We used TempEst v1.5.3 [18] to assess the temporal signal of the datasets and identify-and-remove temporal outliers, defined as sequences that deviate more than two times the interquartile range of the residuals' root-to-tip regression distribution. After these quality control steps, the number of assembled sequences was as follows: DENV1-I (*n*=736 sequences), DENV2-III (*n*=941 sequences), DENV3-III (*n*=755 sequences) and DENV4-II (*n*=283 sequences). Visual inspection of our ML trees identified 9 phylogenetic clades (*n*=20 sequences) with a majority of sequences from Brazil and supported by SH-aLTR between 74 and 100 (Table SI 5).

We next investigated the spatiotemporal origins of each Brazilian lineage using a Bayesian phylogeographic framework in BEAST v.1.10.5 [19]. We used the HKY+G4 nucleotide substitution model [16,20], an uncorrelated relaxed clock model [21] and a flexible Skygrid demographic tree prior with number of grids corresponding the number of years between the time of the most recent common ancestor (TMRCA) estimated using TemPest [22]. We used the new Hamiltonian Monte Carlo Skygrid operator [23] and ML starting trees. All other operators and priors were set as default. For each dataset, we performed at least two Markov Chain Monte Carlo (MCMC) runs of 200 million generations sampling every 20,000 steps with BEAGLE v4 [24] library to enhance computational speed. We used Tracer v1.7.1 [25] to inspect mixing and convergence of chains (effective sample size > 200 for all parameters). Following previous work [26], we used Logcombiner v1.10.5 [19] to obtain a sample of 1,000 empirical dated trees from each dataset. We then estimated time-scaled geographically annotated trees under an asymmetric discrete phylogeographic model [27]. Two spatial discrete traits were reconstructed: Brazilian region (*n=5*) and Brazilian state (*n*=27). A robust counting approach was used to estimate the number and directionality of location-exchange transitions inferred along the posterior distribution of dated trees [28–30]. MCMC chains for phylogeographic analyses were run 50 million generations, sampling every 5,000 steps. We used TreeAnnotator v1.10.5 [19] to infer maximum clade credibility summary trees. The results are sumamrised in Table SI 5 with the XML and summary maximum clade credibility tree files used for the phylogenetic analyses available in the Github repository (https://github.com/obrady/DenSpread_public).

| DENV Serotype | DENV Lineage (n>20) | DENV Lineage Size | | tMRCA of DENV lineage | | | Location (region/state) of DENV lineage | | | | Temporal range of DENV lineages | | DENV Lineage Phylogenetic Support | |
|---|---|---|---|---|---|---|---|---|---|---|---|---|---|---|
| | | n (Brazil) | n (total) | Median | Lower | Upper | Region of intro | PP region | State of intro | PP state | Oldest tip (Brazil) | Youngest tip (Brazil) | PP node | SH-aLRT |
| DENV1 | BR1 (Genotype I) | 48 | 48 | 1983-09-29 | 1982-01-01 | 1985-02-16 | Southeast | 0.9933 | Rio de Janeiro | 0.9826 | 1986 | 2002 | 1 | 99.4 |
| | BR2 (Genotype I) | 89 | 91 | 1998-01-11 | 1996-03-29 | 1998-12-22 | North | 0.9994 | Roraima | 0.9988 | 2000 | 2019 | 1 | 100 |
| | BR3 (Genotype I) | 60 | 61 | 2005-12-30 | 2005-05-25 | 2006-08-02 | North | 0.9923 | Roraima | 0.9904 | 2007 | 2019 | 0.1729 | 100 |
| | BR4 (Genotype I) | 81 | 87 | 2009-05-02 | 2008-10-23 | 2009-11-09 | Southeast | 0.9559 | Rio de Janeiro | 0.9551 | 2010 | 2019 | 1 | 74 |
| DENV2 | BR1 (Genotype III) | 48 | 52 | 1989-02-02 | 1988-05-11 | 1989-08-02 | Southeast | 0.9323 | Rio de Janeiro | 0.9643 | 1990 | 2006 | 1 | 99.9 |
| | BR2 (Genotype III) | 124 | 153 | 2005-03-27 | 2004-11-09 | 2005-07-17 | Northeast | 0.68 | Piaui | 0.998 | 2006 | 2019 | 1 | 93.7 |
| | BR3 (Genotype III) | 237 | 237 | 2013-07-28 | 2012-09-20 | 2014-04-07 | Southeast | 0.9722 | Minas Gerais | 0.9066 | 2016 | 2020 | 1 | 100 |
| DENV3 | BR1* (Genotype III) | 99 | 104 | 1999-10-21 | 1999-04-27 | 2000-02-21 | Northeast | 0.7423 | Pernambuco | 0.9996 | 2001 | 2009 | 0.9948 | 99.8 |
| DENV4 | BR1 (Genotype II) | 182 | 193 | 2009-09-23 | 2008-08-28 | 2010-05-26 | Southeast | 0.57 | Roraima | 0.8762 | 2010 | 2015 | 0.9698 | 100 |

**Table SI 5. - Origins of dengue spread in Brazil from phylogenetic analyses of dengue virus sequence data.** Summary of Bayesian phylogeographic results, assuming region and state as discrete traits (clades with more than 20 sequences). DENV = dengue virus, tMRCA = time to most recent common ancestor, PP = posterior probability, SH-aLRT = Shimodaira–Hasegawa approximate likelihood ratio test.

## 1.4 Historical and future projection of climate and mobility features

Each of the observed climate features included in our analysis were only available for the time period 2000-2015 (TCB and TCW 2000-2014, Land use 2002-2012) which covered the majority of years of the dengue datasets in Mexico and Brazil but was unsuitable for historical reconstruction or future projection purposes. We therefore project expected values of each of these features before 2000 and after 2015 based on the national trend in the most relevant matched variable included in the Tier-1 CMIP6 future projection scenarios[31] (Table SI 6). This approach was not intended as a highly accurate projection of each of the variables used in our study, but to obtain a reasonable range of projected values that are in keeping with the trends in contemporary and projected climate variables.

We obtained the mean predictions from five general circulation models (gfdl-esm4, ipsl-cm6a-lr, mpi-esm1-2-hr, mri-esm2-0, ukesm1-0-ll) under representative concentration pathway (RCP) scenario 3.70 for the variables temperature, humidity and precipitation (all daily min, mean and max) from[31]. The Malaria Atlas Project (MAP) features used in our main analysis were paired with the most relevant RCP features (Table SI 6).

| **Contemporary features** | **RCP feature to assume future trend from** |
|---|---|
| Mean night time temperature | Annual mean monthly minimum temperature |
| SD daytime temperature | Annual standard deviation of monthly maximum temperature |
| Mean day time temperature | Annual mean monthly mean temperature |
| SD EVI | Annual standard deviation of monthly mean humidity |
| Mean EVI | Annual mean of monthly mean humidity |
| SD night time temperature | Annual standard deviation of monthly minimum temperature |
| SD TCW | Annual standard deviation of monthly mean precipitation |

**Table SI 6. Chosen features to match trend for future projection.** RCP = Representative Concentration Pathway. SD = Stand Deviation. EVI = Enhanced Vegetation Index, TCW = Tasselled Cap Wetness.

Annual mean values for Mexico and Brazil were then extracted from all feature rasters for each calendar year. A generalised linear model was then fit for each variable and country with fixed effects for year (linear trend), data source (MAP or RCP) in addition to an intercept. This allowed the between-year trend in the RCP feature values to inform projected past and future MAP feature values despite having different numeric values for some variables. The fit of linear models fit to MAP features only, RCP features only and all data combined (and predicted for MAP or RCP data types) showed a consensus on direction and magnitude of nearly all variables, with the exception of mean daytime temperature in Mexico for which models fit to MAP data suggest decreasing values while RCP data suggests increasing values. Given the limited timespan of the MAP data and a general consensus that temperature is likely to increase over time we rely on the predictions of the model fit to both datatypes.

We then use the trend identified in the models fit to RCP and MAP data (predicting for MAP data values) to project past and present values of each feature. For future values we use the raw RCP predictions plus the coefficient estimate for the RCP datatype in the RCP+MAP model. This aligns RCP datapoints to the scale of the MAP data but retains the between-year variation estimated by the GCMs. To capture appropriate between-year variation in historical feature values we first predict a linear trend from the RCP+MAP model, then add additional randomly sampled noise based on a random sample without replacement of the RCP+MAP model residuals. The resultant projected values for each feature are shown in SI Figure 7.

The values in SI Figure 7 were then used to generate variable, year and country-specific scalar values that informed how each variable changed pre-2000 and post 2015. The feature raster for the year 2005 (the year closest to the average of 2000-2015 conditions) was then multiplied by this scalar value to estimate historical and future trends in each feature.

Projection of mobility variables followed a similar approach. At aggregate levels, movement volumes correlate with population growth and growth in income[32]. We obtained annual national total population estimates and projections from the United Nations World Population prospects 2022[33] and annual national Gross Domestic Product (GDP) estimate and projections from the Organisation for Economic Co-operation and Development (OECD) GDP long term forecast[34]. We converted each indicator into a relative proportionate change since the year 2010 then took an annual average between indicators to provide a year-specific multiplier to increase or decrease human movement variables by. This approach does not account for specific year-on-year changes in mobility nor account for sub-national differences in the rate of change over time, but is intended to provide a rough approximation of changing travel volumes over time and their realistic impact on future trends of dengue spread.

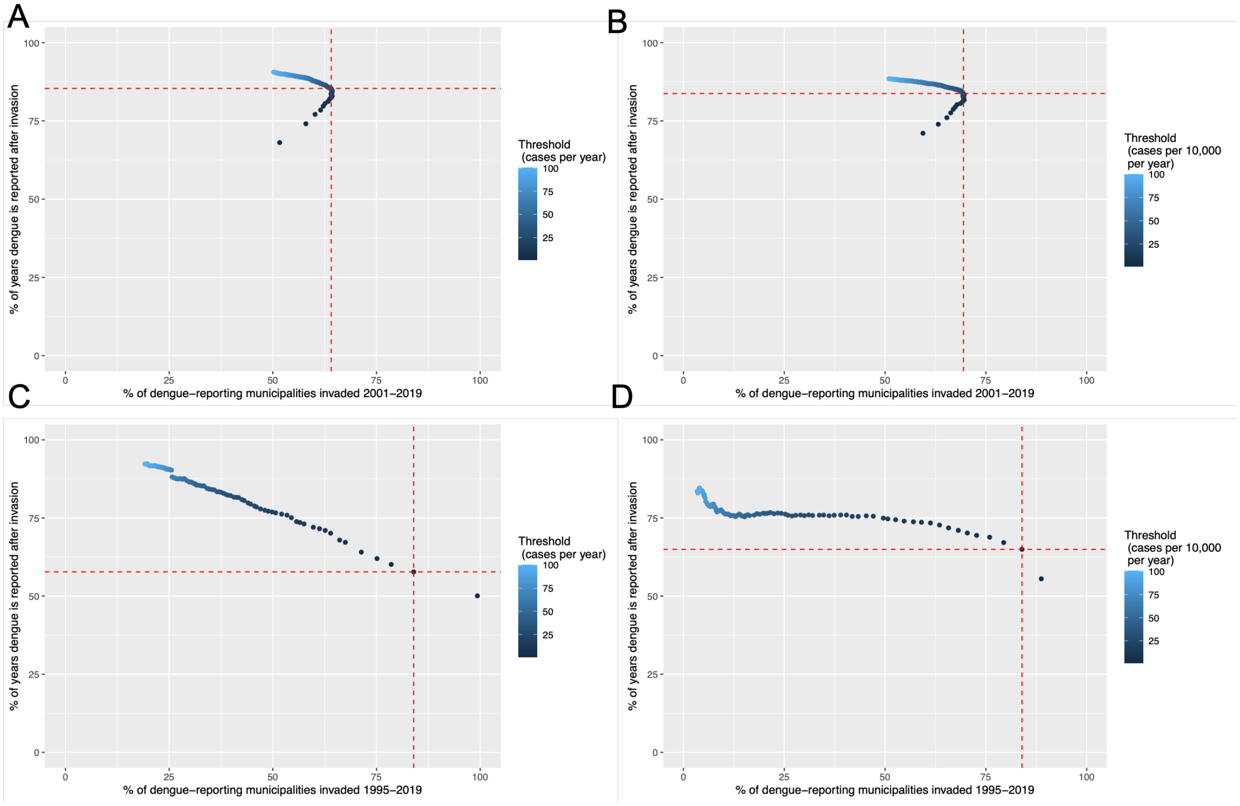


# SI Figure 1 – Optimal thresholds for defining invasion.

Plots show the trade-off between persistence after invasion (y-axis) and proportion of municipalities defined as invaded (x-axis) for different case-based (A and C) and incidence-based (B and D) thresholds for Brazil (A and B) and Mexico (C and D). Dotted red lines show the persistence and proportion invaded for the most optimal point.

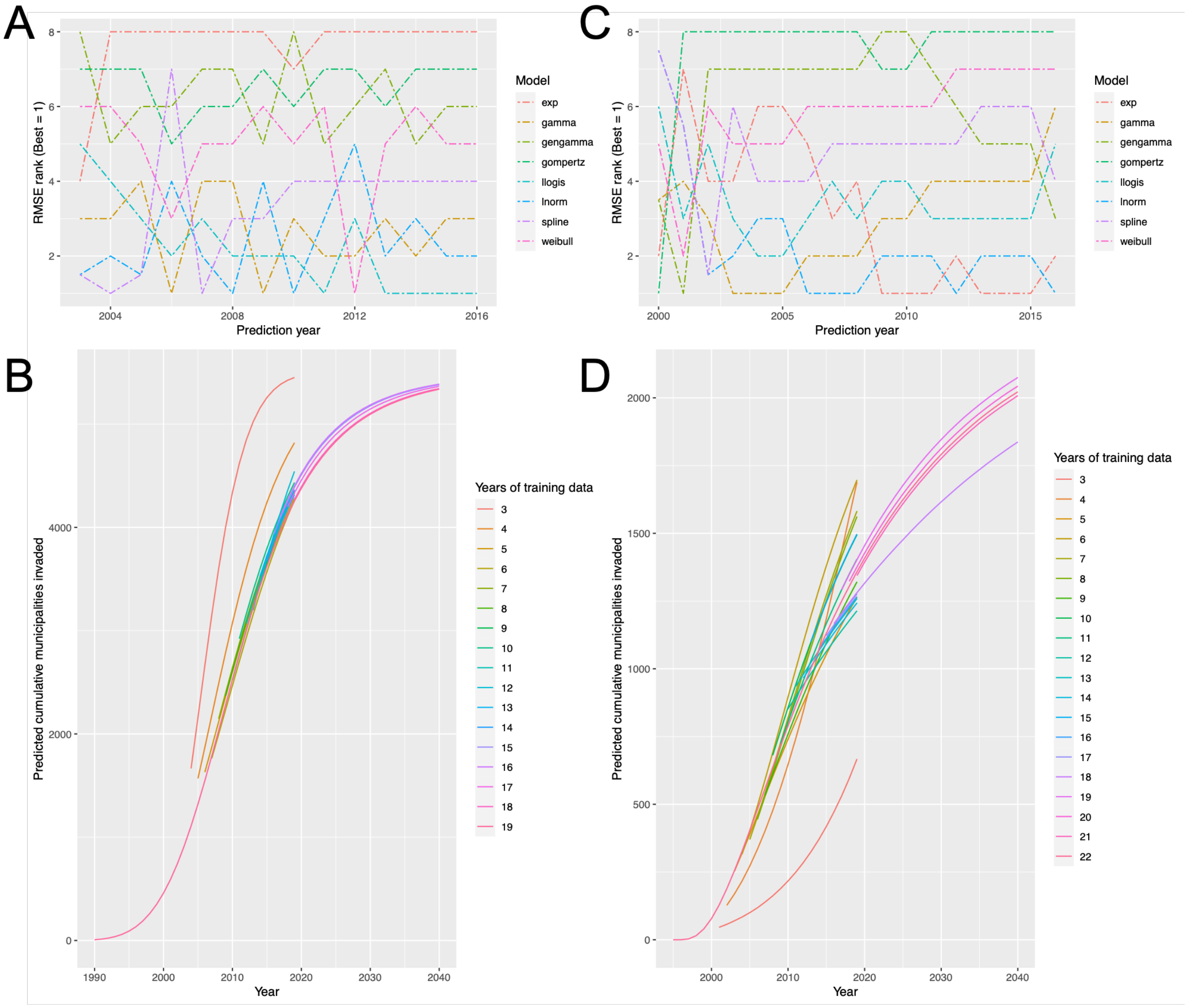


## SI Figure 2- Expanding window time-series cross validation of the survival model in Brazil (A and B) and Mexico (C and D).

Plots A and B show the rank of survival models fit with different functional forms (exp = Exponential, gengamma = Generalised Gamma, llogis = Log-Logistic, lnorm = Log-Normal, spline = 1 knot spline) to data prior to the prediction year and evaluated against the prediction year and all subsequent years, as evaluated by Root Mean Squared Error (RMSE, rank 1 = best fitting). Plots B and D show the risk predictions of the best fitting survival model for each prediction year (only models with at least 3 years of evaluation data shown). Only predictions for the years after the fitting data are shown. Consistent with the future projection analysis, predictions for 2020 onwards are only shown for the five models fitted to the most years of data. Predictions for all years are shown for model fit to all of the training data as it is used for both historical reconstruction and future projection.

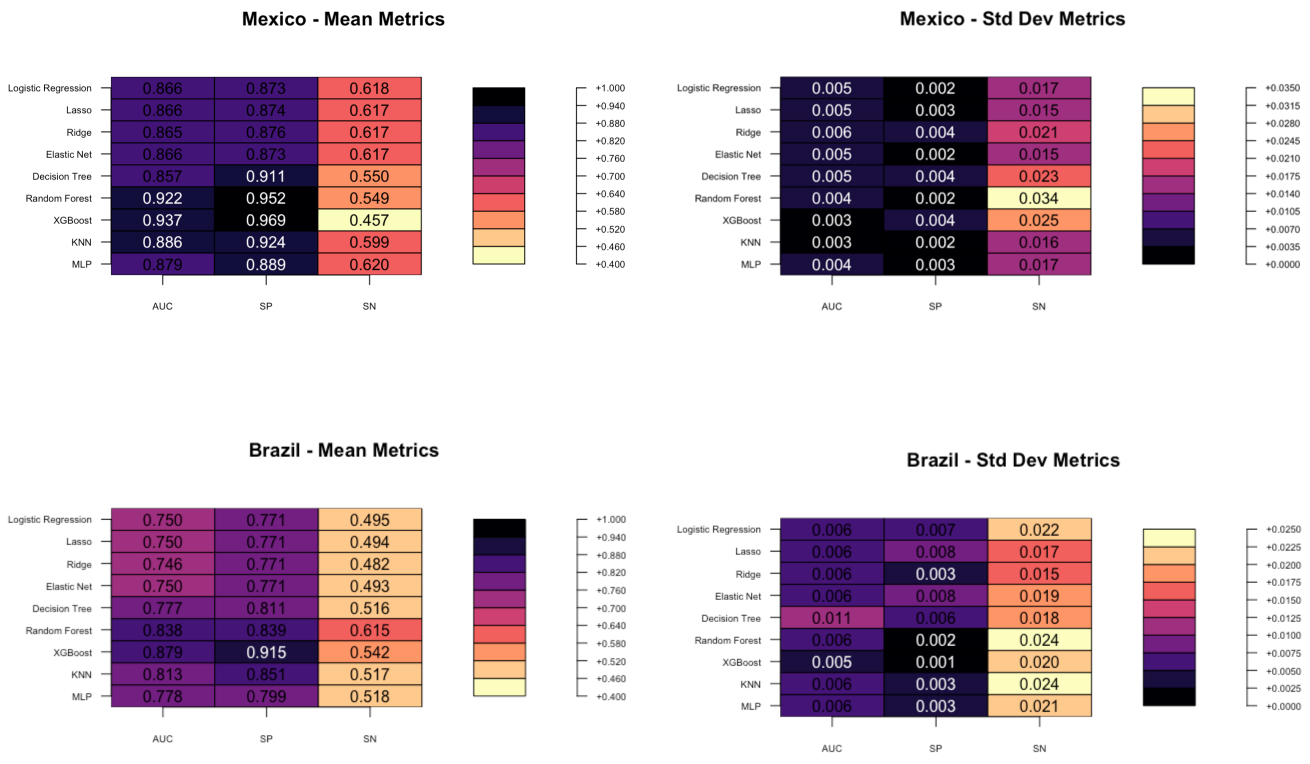


## SI figure 3 – Geospatial model selection heatmap.

Values and colours show the values of sensitivity (SN), specificity (SP) and area under the receiver operating characteristic curve (AUC). All metrics range between 0-1 with higher values (darker colours) indicating superior model predictive performance. Each model was tested over the same 5 random seeds, with the training set (75% of full dataset) split into five-folds for cross-validation for hyperparameter tuning. Classification thresholds (between 0 and 0.99 by increments of 0.01) were set to minimize the difference between specificity and sensitivity in the training set.

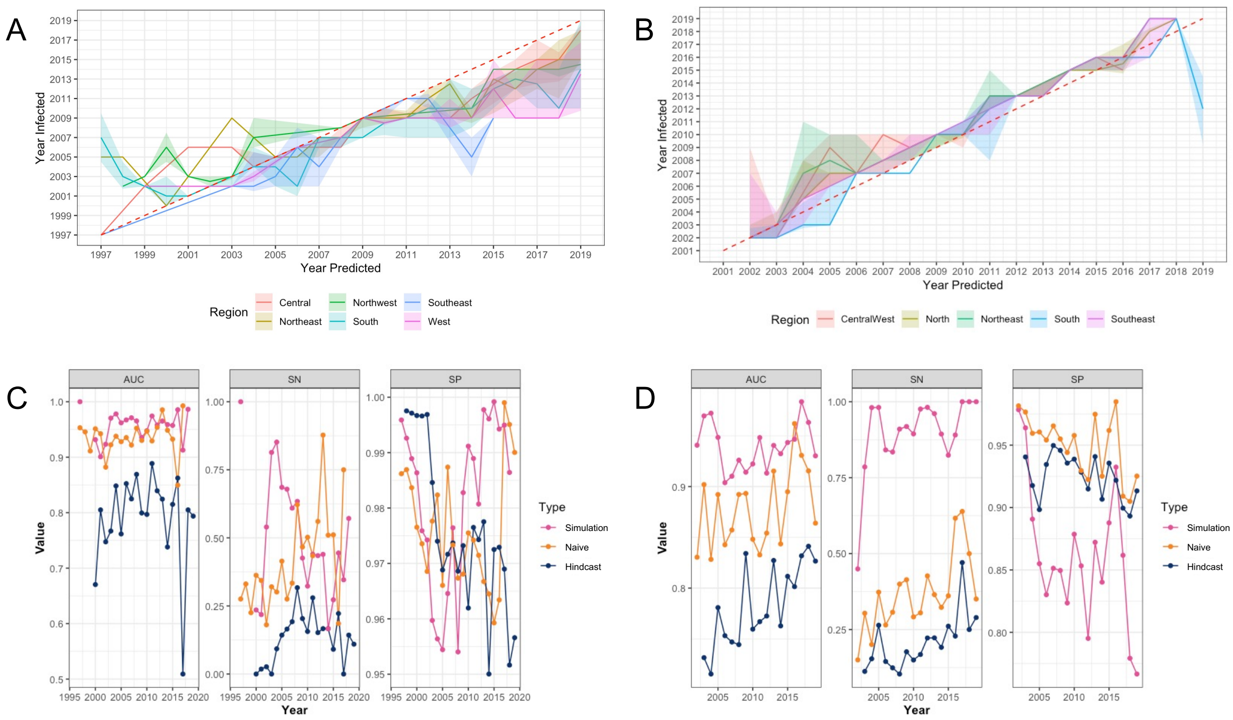


## SI Figure 4 Geospatial model predictive performance over time.

Panels A and B show the correlation between predicted and observed year of arrival of dengue for Mexico (A) and Brazil (B) based on a combined temporal and geospatial model fit to data from all years but only initialised using infected municipalities from 1996 (Mexico) or 2001 (Brazil) (Simulation). Solid lines show the median prediction with shaded areas ndicating the interquartile range of the predicted year of invasion. Figures C and D show the yearly Area Under the (receiver operating characteristic) Curve (AUC), Sensitivity (SN) and Specificity (SP) for the Simulation model, a naïve repeated 75-25 train test split of the data (Naïve) and an expanding window time series cross validation where observed infection sources for the year t – 1 are given to the model when predicting for year t (Hindcast).

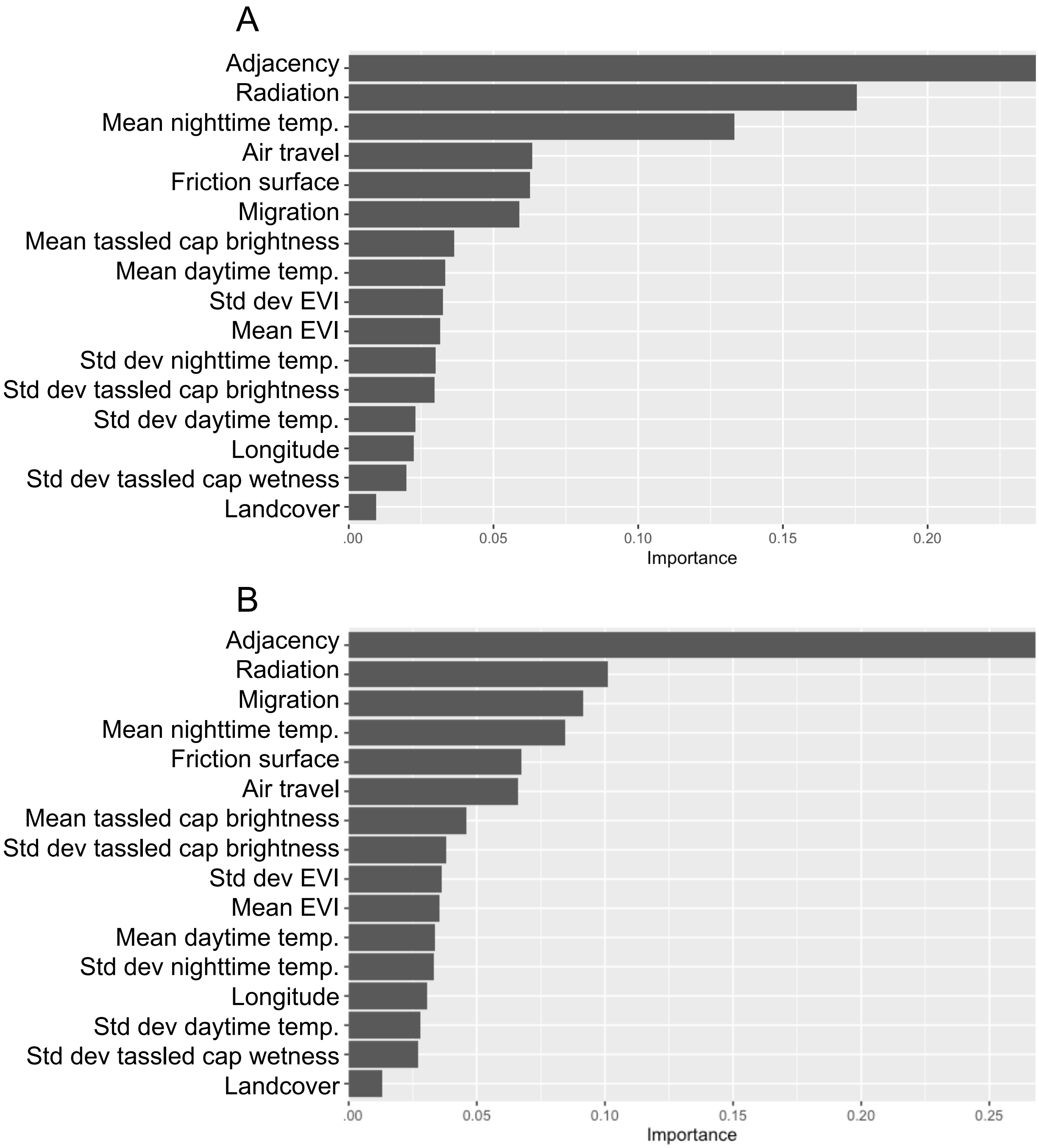


## SI Figure 5 – Variable importance

Plots show the average number of times each feature (row) is selected in to split the dat ain XGBoost models for Mexico (A) and Brazil (B). Variable importance was assessed using XGBoost models fit to data from all years. Temp. = temperature, Std dev = Standard deviation, EVI = environmental vegetation index.

**A**

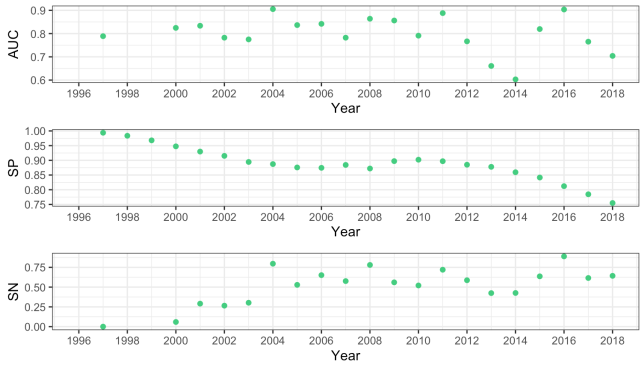


**B**

| Metric | Simulation model | Country cross-validation simulation model |
|---|---|---|
| **AUC** | 0.958 | 0.800 |
| **SP** | 0.981 | 0.888 |
| **SN** | 0.507 | 0.514 |

**C**

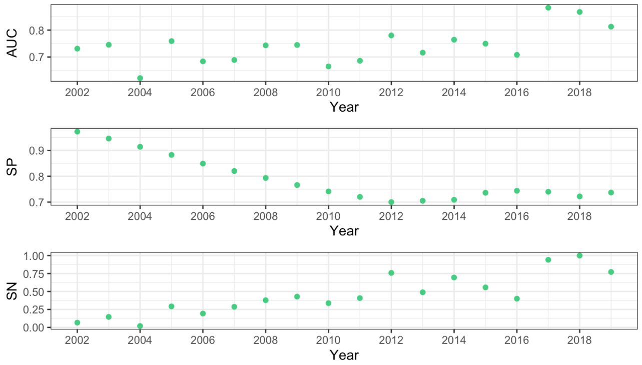


**D**

| Metric | Simulation model | Country cross-validation simulation model |
|---|---|---|
| **AUC** | 0.939 | 0.742 |
| **SP** | 0.862 | 0.789 |
| **SN** | 0.896 | 0.454 |

## SI Figure 6 – Geospatial model country cross-validation.

Panel A shows year-over-year performance metrics when the geospatial model is trained on all spread data from Brazil, initialised with observed source locations in Mexico in 1996, then tested on Mexico spread data 1997-2019. Panel B shows year-over-year performance metrics when the geospatial model is trained on all spread data from Mexico, initialised with observed source locations in Brazil in 2001, then tested on Brazil spread data 2002-2019. Tables B and D summarise the mean annual performance between the default simulation model and country cross validated geospatial models for Mexico and Brazil respectively. AUC = Area Under the Curve, SP= specificity, SN = sensitivity.

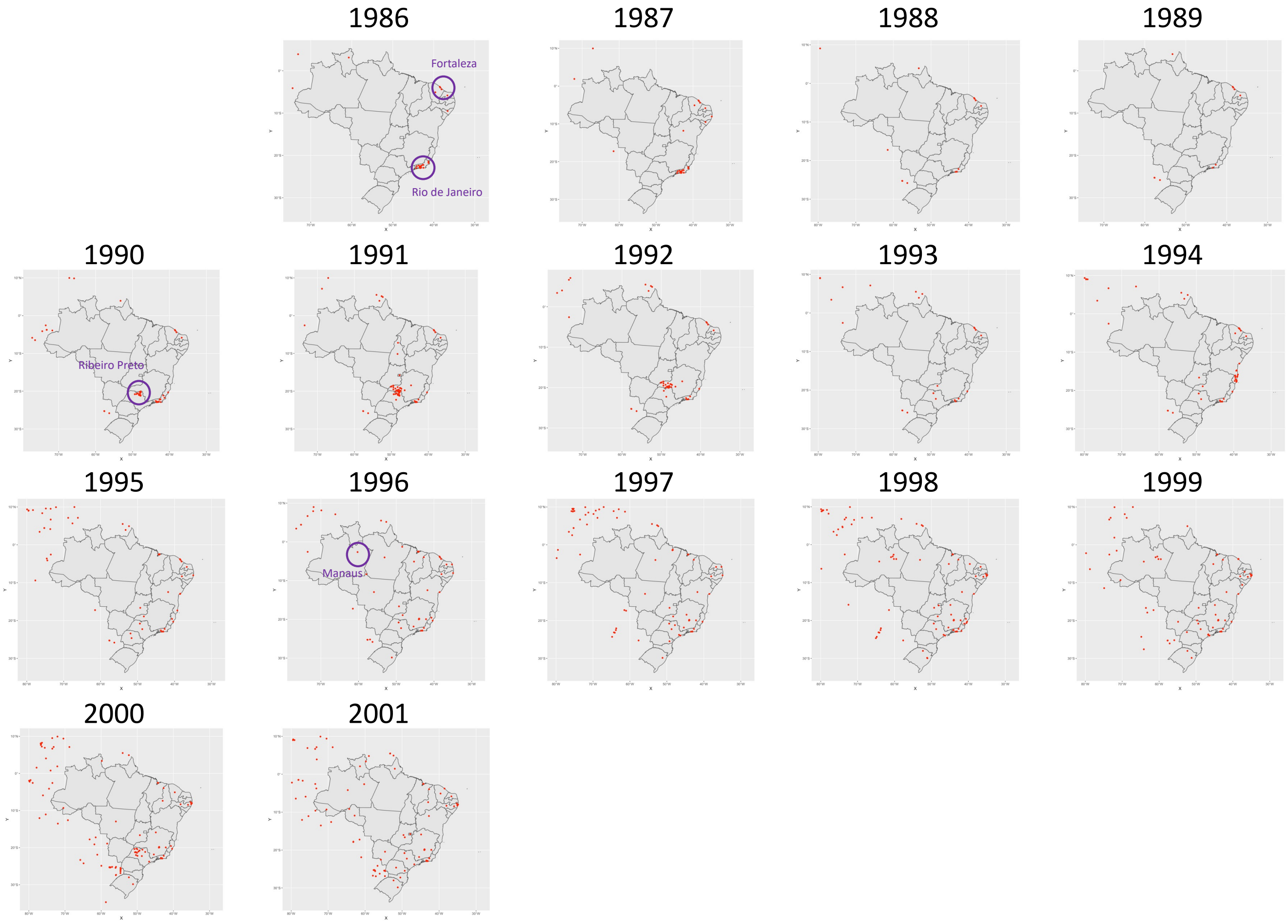


## SI Figure 7 – Possible origins of dengue spread in Brazil from sporadic outbreak reports.

Red dots show reported dengue occurrences in each year within Brazil and border areas of neighbouring countries. Purple circles show the potential origins of spread from case data used in this analysis. Data from Messina et al.[16].

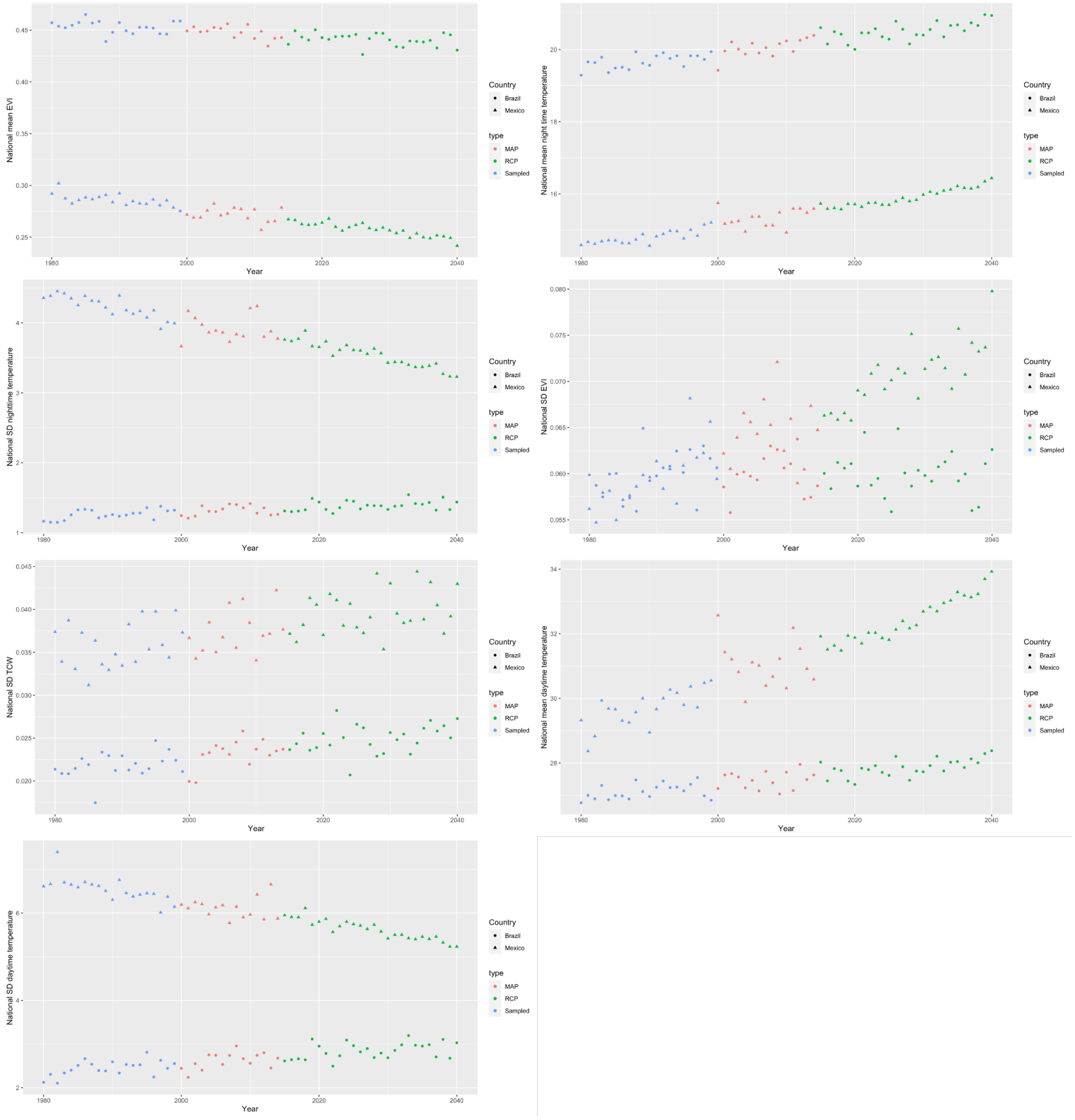


# SI Figure 8 – Projection of model features in keeping with climate scenario.

EVI = Enhanced Vegetation Index, TCW = Tasselled Cap Wetness. MAP = Malaria atlas project covariates, RCP = Representative concentration pathway covariates.

## References


1 Tizzoni M, Bajardi P, Decuyper A, *et al.* On the Use of Human Mobility Proxies for Modeling Epidemics. *PLoS Comput Biol* 2014; **10**: 1003716.

2 Kraemer MUG, Golding N, Bisanzio D, *et al.* Utilizing general human movement models to predict the spread of emerging infectious diseases in resource poor settings. *Sci Rep* 2019; **9**: 1–11.

3 Kraemer MUG, Reiner RC, Brady OJ, *et al.* Modelling the past and future spread of the arbovirus vectors *Aedes aegypti* and *Aedes albopictus*. *Nat Microbiol* 2019; **4**: 854–63.

4 Huber C, Watts A, Grills A, *et al.* Modelling airport catchment areas to anticipate the spread of infectious diseases across land and air travel. *Spat Spatiotemporal Epidemiol* 2021; **36**. DOI:10.1016/J.SSTE.2020.100380.

5 WorldPop. Estimated internal human migration flows between subnational administrative units for malaria endemic countries. 2016. https://www.worldpop.org/geodata/summary?id=1283 (accessed Jan 13, 2021).

6 Weiss DJ, Nelson A, Gibson HS, *et al.* A global map of travel time to cities to assess inequalities in accessibility in 2015. *Nature* 2018; **553**: 333–6.

7 Weiss DJ, Nelson A, Vargas-Ruiz CA, *et al.* Global maps of travel time to healthcare facilities. *Nat Med 2020 2612* 2020; **26**: 1835–8.

8 Messina JP, Brady OJ, Pigott DM, Brownstein JS, Hoen AG, Hay SI. A global compendium of human dengue virus occurrence. *Sci Data* 2014; **1**: 140004.

9 Osanai CH, Travassos da Rosa AP, Tang AT, do Amaral RS, Passos ADC, Tauil PI. Surto de dengue em Boa Vista, Roraima. *Rev Inst Med Trop Sao Paulo* 1983; **25**: 53–4.

10 Nunes M, Faria N, Vasconcelos H, *et al.* Phylogeography of Dengue Virus Serotype 4, Brazil, 2010–2011. *Emerg Infect Dis* 2012; **18**: 1858–64.

11 Clark K, Karsch-Mizrachi I, Lipman DJ, Ostell J, Sayers EW. GenBank. *Nucleic Acids Res* 2016; **44**: D67.

12 Li H. New strategies to improve minimap2 alignment accuracy. *Bioinformatics* 2021; **37**: 4572–4.

13 Jackson B. gofasta: command-line utilities for genomic epidemiology research. *Bioinformatics* 2022; **38**: 4033–5.

14 Minh BQ, Schmidt HA, Chernomor O, *et al.* IQ-TREE 2: New Models and Efficient Methods for Phylogenetic Inference in the Genomic Era. *Mol Biol Evol* 2020; **37**: 1530–4.

15 Tavare S. Some probabilistic and statistical problems in the analysis of DNA sequences. *Some Math Quest Biol / DNA Seq Anal Ed by Robert M Miura* 1986. DOI:10.3/JQUERY-UI.JS.

16 Yang Z. Maximum likelihood phylogenetic estimation from DNA sequences with variable rates over sites: Approximate methods. *J Mol Evol 1994 393* 1994; **39**: 306–14.

17 Guindon S, Dufayard JF, Lefort V, Anisimova M, Hordijk W, Gascuel O. New Algorithms and Methods to Estimate Maximum-Likelihood Phylogenies: Assessing the Performance of PhyML 3.0. *Syst Biol* 2010; **59**: 307–21.

18 Rambaut A, Lam TT, Carvalho LM, Pybus OG. Exploring the temporal structure of heterochronous sequences using TempEst (formerly Path-O-Gen). *Virus Evol* 2016; **2**. DOI:10.1093/VE/VEW007.

19 Suchard MA, Lemey P, Baele G, Ayres DL, Drummond AJ, Rambaut A. Bayesian

phylogenetic and phylodynamic data integration using BEAST 1.10. *Virus Evol* 2018; **4**. DOI:10.1093/VE/VEY016.

20 Hasegawa M, Kishino H, Yano T aki. Dating of the human-ape splitting by a molecular clock of mitochondrial DNA. *J Mol Evol* 1985; **22**: 160–74.

21 Drummond AJ, Ho SYW, Phillips MJ, Rambaut A. Relaxed Phylogenetics and Dating with Confidence. *PLOS Biol* 2006; **4**: e88.

22 Gill MS, Lemey P, Faria NR, Rambaut A, Shapiro B, Suchard MA. Improving Bayesian population dynamics inference: a coalescent-based model for multiple loci. *Mol Biol Evol* 2013; **30**: 713–24.

23 Baele G, Gill MS, Lemey P, *et al.* Hamiltonian Monte Carlo sampling to estimate past population dynamics using the skygrid coalescent model in a Bayesian phylogenetics framework. *Wellcome Open Res 2020 553* 2020; **5**: 53.

24 Ayres DL, Darling A, Zwickl DJ, *et al.* BEAGLE: An Application Programming Interface and High-Performance Computing Library for Statistical Phylogenetics. *Syst Biol* 2012; **61**: 170–3.

25 Rambaut A, Drummond AJ, Xie D, Baele G, Suchard MA. Posterior Summarization in Bayesian Phylogenetics Using Tracer 1.7. *Syst Biol* 2018; **67**: 901–4.

26 Faria NR, Rambaut A, Suchard MA, *et al.* The early spread and epidemic ignition of HIV-1 in human populations. *Science (80- )* 2014; **346**: 56–61.

27 Lemey P, Rambaut A, Drummond AJ, Suchard MA. Bayesian Phylogeography Finds Its Roots. *PLOS Comput Biol* 2009; **5**: e1000520.

28 O'Brien JD, Minin VN, Suchard MA. Learning to Count: Robust Estimates for Labeled Distances between Molecular Sequences. *Mol Biol Evol* 2009; **26**: 801–14.

29 Minin VN, Suchard MA. Counting labeled transitions in continuous-time Markov models of evolution. *J Math Biol 2007 563* 2007; **56**: 391–412.

30 Minin VN, Suchard MA. Fast, accurate and simulation-free stochastic mapping. *Philos Trans R Soc B Biol Sci* 2008; **363**: 3985.

31 Gidden MJ, Riahi K, Smith SJ, *et al.* Global emissions pathways under different socioeconomic scenarios for use in CMIP6: A dataset of harmonized emissions trajectories through the end of the century. *Geosci Model Dev* 2019; **12**: 1443–75.

32 Profillifis V, Botzonis G. Modeling of Transport Demand: Analyzing, Calculating, and Forecasting. Elsevier, 2018.

33 United Nations Department of Economic and Social Affairs Population Division. World Population Prospects 2022. 2022. https://population.un.org/wpp/Download/Standard/MostUsed/ (accessed July 21, 2023).

34 OECD. GDP long-term forecast (indicator). 2018. DOI:10.1787/d927bc18-en.
