# Supplementary material for: Human movement and environmental barriers shape the emergence of dengue
Source: Nat Commun. 2024 May 28;15:4205. doi: 10.1038/s41467-024-48465-0 (PMC11133396; doi:10.1038/s41467-024-48465-0)
Supplement: Supplementary file 3 — Description of Additional Supplementary Files [file 41467_2024_48465_MOESM3_ESM.docx]

File Name: Supplementary Video 1

Description: Predicted invaded municipality by year for Mexico

File Name: Supplementary Video 2

Description: Predicted invaded municipality by year for Brazil
