# Supplementary material for: Human movement and environmental barriers shape the emergence of dengue
Source: Nat Commun. 2024 May 28;15:4205. doi: 10.1038/s41467-024-48465-0 (PMC11133396; doi:10.1038/s41467-024-48465-0)
Supplement: Supplementary file 6 — Reporting Summary [file 41467_2024_48465_MOESM6_ESM.pdf]

nature portfolio

Corresponding author(s): Oliver J Brady

Last updated by author(s): Mar 26, 2024

Reporting Summary

Nature Portfolio wishes to improve the reproducibility of the work that we publish. This form provides structure for consistency and transparency in reporting. For further information on Nature Portfolio policies, see our Editorial Policies and the Editorial Policy Checklist.

Statistics

For all statistical analyses, confirm that the following items are present in the figure legend, table legend, main text, or Methods section.

n/a	Confirmed	
☐	☒	The exact sample size (*n*) for each experimental group/condition, given as a discrete number and unit of measurement
☐	☒	A statement on whether measurements were taken from distinct samples or whether the same sample was measured repeatedly
☐	☒	The statistical test(s) used AND whether they are one- or two-sided<br>*Only common tests should be described solely by name; describe more complex techniques in the Methods section.*
☐	☒	A description of all covariates tested
☐	☒	A description of any assumptions or corrections, such as tests of normality and adjustment for multiple comparisons
☐	☒	A full description of the statistical parameters including central tendency (e.g. means) or other basic estimates (e.g. regression coefficient) AND variation (e.g. standard deviation) or associated estimates of uncertainty (e.g. confidence intervals)
☐	☒	For null hypothesis testing, the test statistic (e.g. *F*, *t*, *r*) with confidence intervals, effect sizes, degrees of freedom and *P* value noted<br>*Give P values as exact values whenever suitable.*
☐	☒	For Bayesian analysis, information on the choice of priors and Markov chain Monte Carlo settings
☐	☒	For hierarchical and complex designs, identification of the appropriate level for tests and full reporting of outcomes
☐	☒	Estimates of effect sizes (e.g. Cohen's *d*, Pearson's *r*), indicating how they were calculated

Our web collection on statistics for biologists contains articles on many of the points above.

Software and code

Policy information about availability of computer code

Data collection	No new data were collected as part of this study. All analyses were on secondary data with software and code used described in the data analysis section
Data analysis	All analyses were performed in the computing software R (version 4.0.1)71 and Rstudio72 (v2022.12.0) using the following packages: geosphere73 (v1.5-18), spdep74 (v1.2-7), tidyverse75 (v1.3.0), Hmisc60 (v4.7-2), tmap76 (v3.3), tidymodels61 (v0.1.0), flexsurv62 (v2.2.0), SHAPforxgboost77 (v0.1.0). An archived version of all the code used for in this paper is available in the following Zenodo repository: https://doi.org/10.5281/zenodo.10890182. Larger processed variable files for environmental and human movement datasets have been deposited in the following Figshare repository: https://doi.org/10.6084/m9.figshare.22047905.v2

For manuscripts utilizing custom algorithms or software that are central to the research but not yet described in published literature, software must be made available to editors and reviewers. We strongly encourage code deposition in a community repository (e.g. GitHub). See the Nature Portfolio guidelines for submitting code & software for further information.

Data

Policy information about availability of data

All manuscripts must include a data availability statement. This statement should provide the following information, where applicable:

- Accession codes, unique identifiers, or web links for publicly available datasets
- A description of any restrictions on data availability
- For clinical datasets or third party data, please ensure that the statement adheres to our policy

Dengue case count data is publicly accessible for Brazil from the Ministério da Saúde's DATASUS system (http://tabnet.datasus.gov.br/cgi/deftohtm.exe?sinanwin/cnv/denguebr.def , http://tabnet.datasus.gov.br/cgi/tabcgi.exe?sinannet/cnv/denguebr.def , http://tabnet.datasus.gov.br/cgi/deftohtm.exe?sinannet/cnv/denguebbr.def) and is avaialble for Mexico from the Sistema de Vigilancia Epidemiológica de Dengue which can be accessed by contacting the Instituto Nacional de Transparencia (https://home.inai.org.mx). Processed versions of both datasets are provided in the study repository. Dengue virus sequence data was obtained from GenBank/NCBI (https://www.ncbi.nlm.nih.gov/genbank/). Climate, environmental and surface travel time covariates are freely available from the Malaria Atlas Project (https://data.malariaatlas.org/maps) and can be downloaded via Google Earth Engine (https://developers.google.com/earth-engine/). High resolution population data and state-level migration estimates can be freely obtained from WorldPop (https://www.worldpop.org). Flight data is not freely available buy can be purchased from IATA (https://www.iata.org). Processed versions of all datasets used in analyses are provided in the study Figshare repository: https://doi.org/10.6084/m9.figshare.22047905.v2 . Source data are provided with this paper.

Research involving human participants, their data, or biological material

Policy information about studies with human participants or human data. See also policy information about sex, gender (identity/presentation), and sexual orientation and race, ethnicity and racism.

Reporting on sex and gender	No analyses were performed on sex or gender because the data avaialble to us did not dissaggregate dengue cases by these variables
Reporting on race, ethnicity, or other socially relevant groupings	No analyses were performed on race, ethnicity, or other socially relevant groupings because the data avaialble to us did not dissaggregate dengue cases by these variables
Population characteristics	All invidiuals residing in Brazil and Mexico
Recruitment	Cases were identified by the national passive surveillance system. In both Mexico and Brazil all citizens and residents have access to the public healthcare system that is capable of diagnosing and reporting dengue cases
Ethics oversight	Not required as the work is a modelling study based only on publicly accessible data

Note that full information on the approval of the study protocol must also be provided in the manuscript.

Field-specific reporting

Please select the one below that is the best fit for your research. If you are not sure, read the appropriate sections before making your selection.

☐ Life sciences ☐ Behavioural & social sciences ☒ Ecological, evolutionary & environmental sciences

For a reference copy of the document with all sections, see nature.com/documents/nr-reporting-summary-flat.pdf

Ecological, evolutionary & environmental sciences study design

All studies must disclose on these points even when the disclosure is negative.

Study description	This is an observational inferential and predictive modelling study that aims to characterise the relationship between several mobility, cliamtic and environmental variables and the arrival time of dengue in each municipality (2nd administrative dividion) in Mexico and Brazil
Research sample	The primary data used in this study is annual counts of dengue cases derived from the national dengue surveillance system in Mexico and Brazil. Cases were restricted to those residing in Brazil (to exclude the small number of infections amng international visitors), but otherwise no exclusion criteria were applied. Because healthcare coverage is universal in both countries and because both public and private facilities are legally required to notify dengue cases when diagnosed, we consider this a representative sample of dengue incidence in these countries
Sampling strategy	No sub-sampling of the national datasets was performed. Because no hypotheses were statistically compared, no sample size calculation was required. Instead we aimed to use the maximum amount of data available over a period where survellance for the diseases was comparable to maximise our chances of characterising the important relationships in our model.
Data collection	Data were downlaoded from the ministry of health data portals (see data accessibility statement).
Timing and spatial scale	For Brazil, annual total dengue cases were extracted for 5570 municipalities for each year for which national data were available (January 2001- December 2019). For Mexico, annual total dengue cases for 2456 municipalities were extracted for each year

	1995-2019. We chose 2nd administrative level as the maximum spatial resolution the data were avaialble at to maximise our chances of characterising spatial spread and chose annual temporal resolution to improve the precision of our definition of invaded / not invaded areas (tested in SI Fig. 1)
Data exclusions	Dengue cases with an address outside of Brazil were excluded from the Brazilian dengue dataset. No other exclusions were applied.
Reproducibility	Results were verified independently by two different authors (VH and OB). Internal cross valdiation experiments (both temporal and spatial) also tested generalisability of the findings.
Randomization	Not relevant to this study as it is an observational study with no intervention. Assignment of exposures was, therefore, already predetermined
Blinding	Blinding was not possible in this study as it is a secondary analysis of observational data. Exposures to be tested were pre-specified prior to data analysis.

Did the study involve field work? ☐ Yes ☒ No

Reporting for specific materials, systems and methods

We require information from authors about some types of materials, experimental systems and methods used in many studies. Here, indicate whether each material, system or method listed is relevant to your study. If you are not sure if a list item applies to your research, read the appropriate section before selecting a response.

Materials & experimental systems

n/a	Involved in the study
☒	☐ Antibodies
☒	☐ Eukaryotic cell lines
☒	☐ Palaeontology and archaeology
☒	☐ Animals and other organisms
☒	☐ Clinical data
☒	☐ Dual use research of concern
☒	☐ Plants

Methods

n/a	Involved in the study
☒	☐ ChIP-seq
☒	☐ Flow cytometry
☒	☐ MRI-based neuroimaging

Plants

Seed stocks	NA
Novel plant genotypes	NA
Authentication	NA
